# The association of weight status and weight perception with number of confidants in adolescents

**DOI:** 10.1371/journal.pone.0225908

**Published:** 2019-12-04

**Authors:** Asuka Nishida, Jerome Clifford Foo, Shinji Shimodera, Atsushi Nishida, Yuji Okazaki, Fumiharu Togo, Tsukasa Sasaki

**Affiliations:** 1 Department of Physical and Health Education, Graduate School of Education, The University of Tokyo, Tokyo, Japan; 2 Department of Genetic Epidemiology in Psychiatry, Central Institute of Mental Health, Medical Faculty Mannheim, University of Heidelberg, Mannheim, Germany; 3 Department of Neuropsychiatry, Kochi Medical School, Kochi University, Kochi, Japan; 4 Department of Psychiatry and Behavioral Science, Tokyo Metropolitan Institute of Medical Science, Tokyo, Japan; 5 Tokyo Metropolitan Matsuzawa Hospital, Tokyo, Japan; Chiba Daigaku, JAPAN

## Abstract

Weight status and self-weight perception are related to social relationship issues. Studies have suggested links between non-normal weight status or weight perception and youths having fewer confidants, but these relationships are unclear and remain to be studied. This preliminary cross-sectional study examined the effects of weight status and weight perception on the number of confidants in adolescents. Self-report data from 15,279 grade 7–12 students (54.2% boys) were analyzed. The number of confidants (0–3 or ≥ 4) was examined, according to five weight status categories (underweight, low-normal weight, mid-normal weight (reference), high-normal weight, overweight, with Body Mass Index corresponding to ≤ 18.5, ≤ 20.0, ≤ 22.5, ≤ 25.0 and > 25.0 in adults, respectively), and five weight perception categories (*too* thin, *a bit* thin, good (reference), *a bit* fat, *too* fat). Boys and girls who were overweight and those who perceived themselves to be *too* fat were significantly more likely to have few confidants. High-normal weight in girls and self-perception of being *a bit* fat in boys were also associated with having few confidants. In boys, underweight and self-perception of being *too* thin were additionally associated with having few confidants. Adolescents with non-normal weight status or weight perception may have fewer confidants and require more social support.

## Introduction

Adolescence is a sensitive developmental period in which youths acquire more sophistication in social interactions and experience changes in social expectations [1]. During adolescence, the prevalence of mental health problems increases sharply [2]. Among these problems are depressive disorders, self-harm, and anxiety disorders, which are some of the top ten leading causes of Disability-Adjusted Life Years [3] lost by adolescents [4]. One factor that protects adolescents from mental health problems is having access to confidants; people to whom these adolescents can talk about their problems. Adolescents who have few or no confidants are more likely to have mental health problems such as higher levels of depressive symptoms [5–7]. It has been reported that having 3 or fewer confidants is associated with the development of mental health issues [8].

Overweight and obese adolescents often face social disadvantages in multiple domains of living, including interpersonal relationships, as a result of weight stigmatization [9]. Weight stigma portrays overweight and obese individuals with negative characteristics and beliefs, such as lazy, stupid, less popular and lacking in friends [9]. Some evidence suggests that during adolescence, being overweight/obese, which is highly stigmatized in our society, may compromise their access to confidants. Overweight/obese adolescents may have smaller social network sizes compared with non-overweight/obese peers. For example, being overweight/obese during adolescence predicts social marginalization [10], including difficulty in making new friends [11,12]. Overweight/obese adolescents are also less frequently nominated by their classmates as friends [11,13,14]. In addition, overweight/obese adolescents often have difficulty perceiving their best friends as confidants [15] or have relationship difficulties with close friends [12]. Overweight adolescents often report low quality of family interactions [16], such as having difficulties in relationships with their parents [12], which may also contribute to their lack of confiding relationships.

On the other hand, some studies have observed that being underweight can be related to having less access to confidants. For example, underweight adolescents are more likely to have problems in social behavior such as getting along with friends [17]. Underweight adolescents also report a low frequency of family interactions, including a low frequency of meals eaten together with family [18]. Furthermore, being underweight in boys has also been significantly associated with having difficulty in discussing personal issues with parents [18]. A meta-analysis which examined adults found that Body Mass Index (BMI) was significantly positively associated with extraversion in males [19].

It is possible that weight perception of self (WP) may also affect confiding relationships in adolescents, regardless of their weight status (WS). A systematic review observed that negative body image (being both too thin and too fat) was often associated with lower levels of extraversion in people of all ages [20]. Previous studies have also observed that adolescents who perceive themselves as either being thin or fat report more social adaptation problems (e.g. problems becoming accustomed to school life) [17,21] and impairment in social functioning (e.g. trouble getting along with peers) [22]. In addition, a study observed that WP was discordant with WS in a quarter of adolescents [23]. Furthermore, the effect of WP may not be in accordance with the effect of the corresponding WS. For example, a perception of being either thin or fat might not affect social relationships as negatively as actually being underweight or overweight might, given that girls may be more likely to become friends with other girls who have similar weight concerns [24].

According to these findings, WS and WP might be associated with the number of confiding relationships in adolescents; these relationships, however, remain to be investigated. The present study examines whether junior and senior high school students with non-normal WS and WP have fewer confidants than their counterparts with normal WS and WP, using a self-report questionnaire.

## Materials and methods

### Populations and procedures

A school-based cross-sectional survey targeting junior and senior high school students (Grades 7–12) was conducted from 2006 to 2009 in Japan. The principal investigators approached the school principals of all public junior and senior high schools in Kochi prefecture (population: 780,000), and all public junior high schools in Tsu City (population: 290,000) in Mie prefecture. School principals were told that participation in the survey was voluntary. The principals then consulted with the teachers. The parents and guardians received a letter from the principal investigators that asked them to notify the school if they withheld consent for their child’s participation in the present research. Among the 138 public junior and 36 public senior high schools, 45 junior high and 28 senior high schools participated in this study. Overall, 19,436 students were asked to participate in the self-reported survey. Of these, 798 were absent on the days of the survey and 388 declined to participate. Of the 18,250 students who agreed to participate, 2,234 students with missing data for height (*n* = 839) or weight (*n* = 2,136) were excluded. Furthermore, data from 175 students were excluded for not meeting a BMI value of 12–40 (*n* = 125) or not being within height threshold of 140–200 cm (*n* = 50) (i.e. invalid responses). Also, data from 416 students were excluded from the analysis because of incomplete answers to questions for WP (*n* = 265) or for the number of confidants (*n* = 162). In the end, data from 15,279 (83.7%; mean age = 15.3 ± 1.7; 54.2% boys) students were analyzed.

On the survey days, the teachers distributed to the students the survey questionnaire and an envelope (in which to seal the completed questionnaire before handing it back to the teachers). The students were told that participation in the study was anonymous and voluntary, and that answers would be kept confidential. Research staff collected the sealed questionnaires at each school. The study was conducted in accordance with Japan’s Ethical Guidelines for Epidemiological Research. The data collection was approved by the ethics committees of the Tokyo Metropolitan Institute of Psychiatry (approval number: 20–9), the Mie University School of Medicine (approval number: 603), and Kochi Medical School at Kochi University (approval number: 20–57).

### Measurements

The questionnaire items and response options used in the present study are shown in S1 Table, including the measurement of independent and dependent variables and covariates in the original language and English. Details of the coding for each variable are described in the following sub-sections.

#### Weight status and weight perception

Self-reported height and weight values were used to calculate BMI (kg/m^2^). BMI values were initially split into four WS categories (underweight, normal weight, overweight, and obese), using BMI cut-off values that are equivalent to adult BMI values of 18.5, 25.0, and 30.0, based on age- and sex-specific cut-off values for underweightedness [25] and overweightedness and obesity [26] in adolescents. The majority of subjects fell in the normal weight category. Thus, we further divided the normal weight category into low-normal weight, mid-normal weight, and high-normal weight using cut-off points which are comparable to the BMI values of 20.0 and 22.5 for adults [27–29]. Finally, the number of divisions for WS was reduced from 6 to 5 groups by merging the obesity group into the overweight group, since the number of obese subjects was small (*n* = 224 in boys; *n* = 67 in girls). In the end, the five WS categories were defined, with cut-off values adjusted for age and sex corresponding to the following equivalent BMIs in adults: Underweight: BMI < 18.5; Low-normal weight: 18.5 ≤ BMI < 20.0; Mid-normal weight: 20.0 ≤ BMI < 22.5; High-normal weight: 22.5 ≤ BMI < 25.0; Overweight: BMI ≥ 25.0.

WP was measured using the following one-item question: “What do you think of your current body weight?” The students were given a five-point Likert scale with responses defined as follows: 1. “Too fat”; 2. “A bit fat”; 3. “Good”; 4. “A bit thin”; and 5. “Too thin”. The students responded to the question by circling one of the given choices. In the analyses, each answer was considered as one category.

#### Number of confidants

The number of confidants was assessed with the following question, “How many people are there for you to confide in about your problems/concerns?”, with five possible answers: 1. “None”; 2. “One”; 3. “Two”; 4. “Three”; 5. “Four or more”. The students responded to the question by circling one of the given choices. Adolescents with 3 or fewer confidants were considered as having few confidants. A dichotomous variable was then created by combining answers of 1 to 4 into the “0–3 (Few) confidants” group, and putting answers of 5 in the “≥ 4 (More) confidants” group.

#### Covariates

Earlier studies found that non-normal WS significantly increases the risk of being bullied [30–32]. In addition, those who have past experience of being bullied often report low levels of support from friends [33], which may imply fewer confidants of the victims of bullying. Also, having few confidants has been reported to be significantly associated with depression and/or anxiety [5–7]. Our study entered these variables into the final analysis as confounders so that the risk of having few confidants in adolescents with non-normal WS/WP is examined regardless of the presence/absence of experience of being bullied and the current status of mental health. Experience of being bullied (yes or no) was assessed by asking the students, “Have you been bullied within the past year?” Depression/anxiety symptoms were measured using the Japanese version of the 12-item General Health Questionnaire (GHQ-12). The validity of GHQ-12 in adolescents has been previously confirmed [34]. In addition, the validity and reliability of the Japanese version of GHQ-12 has been established in people of all ages [35]. While each item of GHQ-12 was rated on a four-point Likert scale, the present study applied a bimodal scoring method (0-0-1-1); the Cronbach’s alpha using this scoring method was 0.84 in the present sample. A score of 4 or higher in the possible score range of 0 to 12 is indicative of the risk of depression/anxiety in Japanese adults and adolescents [36]. These scores were entered as a dichotomous variable which were coded to 0 (scores from 0–3) and 1 (4–12) in the analyses.

### Statistical analysis

The associations of WS or WP with the number of confidants were tested using binary logistic regression. The number of confidants was specified as the dependent variable (Few: 0–3, More: ≥ 4), while either WS or WP was included as an independent variable. Analyses were adjusted for age, experience of being bullied (0 or 1) and GHQ-12 score (0 or 1). Mid-normal weight or perceiving oneself as good was chosen as the reference category in the analyses. All analyses were stratified by sex. In the adjusted analyses, 20 boys and 32 girls were excluded due to missing data about the experience of being bullied; a total of 8,088 boys and 7,139 girls were analyzed. Since age was a continuous variable, the present study conducted the Hosmer-Lemeshow test, a goodness of fit test for logistic regression, to assess how well the data fits the logistic regression models. It gave *p*-values of 0.43 and 0.75 for boys and girls respectively, indicating no evidence of poor fit. Testing the dichotomous variables gave *p*-value of 1. All statistical analyses were conducted using IBM SPSS Statistics version 25.0 for Microsoft Windows (IBM Corp., Armonk, NY).

## Results

### Descriptive statistics

The largest proportion of students (boys: 41.7%, girls: 40.1%) fell into the mid-normal weight group (Table 1). The prevalence of being underweight was 8.7% in boys and 12.7% in girls, while being overweight was 11.4% in boys and 6.6% in girls. WP for boys had a normal distribution, while the distribution of girls was skewed toward the perception of being fat. The distributions of WS, WP, and the number of confidants did not differ substantially across school level (Junior high / Senior high); in both boys and girls, the same category always showed the largest proportion within each variable. The distributions of WS, WP, and the number of confidants, stratified by school level are shown in S1–S4 Tables. As shown in Table 2, the distribution of WP in boys was relatively similar to the distribution for WS. However, a large proportion of girls who were underweight or normal weight reported perceiving themselves as fat. In both boys and girls, the highest prevalence of having 3 or fewer confidants was observed in overweight students.

**Table 1:** Weight status, weight perception, the number of confidants, experience of being bullied, and the General Health Questionnaire (GHQ)-12 scores in boys and girls.

	Boys	Girls
***N***	8,108	7,171
**Age (years), mean ± SD**	15.3 ± 1.7	15.3 ± 1.7
**BMI (kg/m**^**2**^**), mean ± SD**	20.2 ± 3.2	19.9 ± 2.7
**Weight status, *n* (%)**		
Underweight	716 (8.8)	922 (12.9)
Low-normal weight	1,610 (19.9)	1,765 (24.6)
Mid-normal weight	3,402 (42.0)	2,882 (40.2)
High-normal weight	1,456 (18.0)	1,131 (15.8)
Overweight	699 (8.6)	404 (5.6)
Obese	225 (2.8)	67 (0.9)
**Weight perception, *n* (%)**		
*Too* thin	589 (7.3)	87 (1.2)
*A bit* thin	1,610 (19.9)	328 (4.6)
Good	3,291 (40.6)	1,732 (24.2)
*A bit* fat	1,910 (23.6)	3,328 (46.4)
*Too* fat	708 (8.7)	1,696 (23.7)
**Number of confidant(s)**^**a**^**, *n* (%)**		
None	1,844 (22.7)	777 (10.8)
1	744 (9.2)	860 (12.0)
2	1,257 (15.5)	1,420 (19.8)
3	822 (10.1)	1,136 (15.8)
≥ 4	3,441 (42.4)	2,978 (41.5)
**Experience of being bullied (past 1 year), *n* (%)**		
(+)	621 (7.7)	505 (7.0)
**GHQ-12 score, *n* (%)**		
≥ 4	2,699 (33.3)	3,936 (54.9)

*n*, number of subjects; SD, standard deviation.

^a^ How many people are there for you to confide in about your concerns/problems?

**Table 2:** Weight perception and the number of confidants according to weight status, in boys and girls, *n* (%).

	Underweight	Low-normal weight	Mid-normal weight	High-normal weight	Overweight
	**Boys (*N* = 8,108)**				
**Weight perception**					
*Too* thin	239 (33.4)	220 (13.7)	115 (3.4)	11 (0.8)	4 (0.4)
*A bit* thin	262 (36.6)	586 (36.4)	673 (19.8)	78 (5.4)	11 (1.2)
Good	184 (25.7)	648 (40.2)	1,773 (52.1)	590 (40.5)	96 (10.4)
*A bit* fat	24 (3.4)	132 (8.2)	759 (22.3)	636 (43.7)	359 (38.9)
*Too* fat	7 (1.0)	24 (1.5)	82 (2.4)	141 (9.7)	454 (49.1)
**Number of confidants**^**a**^					
0–3	432 (60.3)	941 (58.4)	1,886 (55.4)	843 (57.9)	565 (61.1)
≥ 4	284 (39.7)	669 (41.6)	1,516 (44.6)	613 (42.1)	359 (38.9)
	**Girls (*N* = 7,171)**				
**Weight perception**					
*Too* thin	68 (7.4)	14 (0.8)	4 (0.1)	1 (0.1)	0 (0.0)
*A bit* thin	200 (21.7)	105 (5.9)	22 (0.8)	0 (0.0)	1 (0.2)
Good	408 (44.3)	702 (39.8)	557 (19.3)	58 (5.1)	7 (1.5)
*A bit* fat	214 (23.2)	794 (45.0)	1,681 (58.3)	547 (48.4)	92 (19.5)
*Too* fat	32 (3.5)	150 (8.5)	618 (21.4)	525 (46.4)	371 (78.8)
**Number of confidants**^**a**^					
0–3	528 (57.3)	1,008 (57.1)	1,638 (56.8)	709 (62.7)	310 (65.8)
≥ 4	394 (42.7)	757 (42.9)	1,244 (43.2)	422 (37.3)	161 (34.2)

*n*, number of subjects.

^a^ How many people are there for you to confide in about your concerns/problems?

### Binary logistic regression

The results for the binary logistic regression analyses are summarized in Table 3. Before adjustment for covariates, the odds ratios (ORs) were statistically significant for having few confidants in boys who were underweight (*p* < .05), low-normal weight (*p* < .05) or overweight (*p* < .01) and those who perceived themselves to be *too* thin (*p* < .001), *a bit* fat (*p* < .001), or *too* fat (*p* < .001). In girls, ORs were significant for those who were high-normal weight (*p* < .001) or overweight (*p* < .001) and those who perceived themselves to be *a bit* fat (*p* < .05) or *too* fat (*p* < .001). After adjusting for age, experience of being bullied and GHQ-12 scores, the odds ratios were statistically significant in boys who were underweight (*p* < .05) and overweight (*p* < .05) and those who perceived themselves as being *too* thin (*p* < .05), *a bit* fat (*p* < .01), and *too* fat (*p* < .01). In girls, being overweight (*p* < .001) and high-normal weight (*p* < .01) and perceiving oneself to be *too* fat (*p* < .05) showed significant odds ratios.

**Table 3:** Odds ratios and 95% CIs for the effects of WS and WP on having few confidants when adjusted for age, experience of being bullied, and depression/anxiety symptoms.

	Unadjusted	Adjusted
	**Boys**	
Underweight	**1.22 (1.04–1.44)**	**1.23 (1.04–1.45)**
Low-normal weight	**1.13 (1.00–1.27)**	*1*.*12 (0*.*99*–*1*.*27)*
High-normal weight	1.10 (0.98–1.25)	1.09 (0.96–1.24)
Overweight	**1.26 (1.09–1.47)**	**1.21 (1.04–1.41)**
*Too* thin	**1.37 (1.14–1.64)**	**1.21 (1.01–1.45)**
*A bit* thin	1.03 (0.92–1.17)	1.00 (0.90–1.13)
*A bit* fat	**1.33 (1.19–1.49)**	**1.21 (1.07–1.36)**
*Too* fat	**1.54 (1.30–1.83)**	**1.31 (1.10–1.56)**
	**Girls**	
Underweight	1.01 (0.87**–**1.17)	1.01 (0.86–1.17)
Low-normal weight	1.01 (0.90–1.14)	1.00 (0.89–1.13)
High-normal weight	**1.28 (1.11–1.47)**	**1.26 (1.09–1.46)**
Overweight	**1.45 (1.18–1.78)**	**1.41 (1.15–1.74)**
*Too* thin	1.43 (0.92–2.24)	1.31 (0.83–2.06)
*A bit* thin	1.04 (0.82–1.33)	0.99 (0.78–1.27)
*A bit* fat	**1.15 (1.02–1.29)**	1.05 (0.93–1.18)
*Too* fat	**1.42 (1.24–1.63)**	**1.19 (1.03–1.37)**

CI, confidence interval; WS, weight status (reference: mid-normal weight); WP, weight perception (reference: perceiving one's weight to be good). Significant associations are indicated in bold (*p* < .05) and italic (*p* < .10) letters. Covariates include age, experience of being bullied, and GHQ-12 score (0/1).

According to the sample calculation, the required sample size for the present binary logistic regression was shown to be 2,548 references versus 849 cases, when *α* = .05 and *β* = 0.1, with the assumptions of the proportion of adolescents who have few confidants in the reference group = 0.4, OR = 1.3, and the ratio of the references versus cases = 3:1. When the ratio of the references versus cases is assumed = 1:1, the sample size required is calculated as 1,276 references versus 1,276 cases. The actual sample size, summarized in Table 1, seems to be close to the calculated sample size, except for the groups of “underweight” boys and “overweight (plus obese)” girls among WS categories (note that “obese” and “overweight” were merged into the “overweight” category in the analysis), and the groups of boys and girls perceiving themselves to be “*too* thin”, girls perceiving themselves to be “*a bit* thin”, and boys perceiving themselves to be “*too* fat” among WP categories. Among these groups, no significant associations between WS/WP and the number of confidants were observed in girls perceiving themselves to be “*a bit* thin” and “*too* thin”.

## Discussion

This is the first study to examine the associations of WS and WP with the number of confidants in adolescents. Adolescents having non-normal WS or WP had fewer confidants. Both boys and girls who were overweight, when compared with mid-normal weight counterparts, or perceived themselves as being *too* fat, when compared with those who perceived their weight to be good, were statistically significantly more likely to have few confidants. Girls who were high-normal weight were also more likely to have few confidants, as were boys who were underweight or perceived themselves to be *too* thin or *a bit* fat. Most associations remained significant after controlling for depressive and anxiety symptoms, suggesting that adolescents with non-normal WS or WP, even when they are currently without the symptoms, may be more likely to have few confidants compared with their reference counterparts.

Overweight adolescents being more likely to have few confidants is in line with studies in the literature. A previous study reported that overweight adolescents often do not have close friends or have difficulties in family relationships [12]. Another study found that overweight adolescents often do not perceive their best friends as confidants [15]. One study reported that overweight girls are less likely than normal weight counterparts to perceive their family members as confidants [16]. The reasons for overweight adolescents having fewer confidants may be partly explained by psychosocial factors including stigmatization. Overweight adolescents may often experience social rejection since individuals, including adolescents [37], often prefer thinner people [38]. High stigma with respect to body weight may make overweight adolescents opt out of a broader peer network [39], leading to less time spent with friends after school [12,15,17,22]. Overweight adolescents are also more likely to make friends with overweight peers [40] or peers with similar levels of bulimic behavior [24], which can limit their sources of friendships. Girls who had high-normal weight in the present study were also more likely to have few confidants. High-normal weight girls, as well as overweight girls, may experience social rejection by peers since teenage girls often characterize their ideal weight as low-normal weight [41].

Adolescents who perceived themselves to be *too* fat were more likely to have few confidants. This may be in line with previous studies finding that perceived-overweightedness was associated with social relationship difficulties [17,21] and withdrawnness [17] in adolescents and that perceived-overweightedness could be predicted by receiving negative comments from peers [42]. Similarly, boys with the perception that they were *a bit* fat were also more likely to have few confidants, but this was not observed in girls. A previous study reported that girls are more likely to become friends with other girls who have similar weight/shape concerns [24]. In the present study, more than half of the girls reported perceiving themselves to be *a bit* fat. In boys, the proportion was half of that in girls. This may suggest that the perception of being *a bit* fat may actually be “normal” in girls, mitigating any negative effects on the number of confidants, but not in boys.

The present study also showed that boys who were underweight or perceived themselves to be underweight were more likely to have few confidants. This may be in line with previous findings that males who are underweight are more likely to be introverted [19], and that boys who are extremely thin and who perceive themselves to be underweight have difficulty confiding in parents about personal issues [18]. While girls typically wish to be thinner, in contrast, boys often want to be larger [43]. In boys, it has been shown that lower WS predicted a stronger increase in muscularity concerns, referred to as muscular-ideal internalization, compared with heavier weight counterparts [44]. This desire for muscularity might have confounded the present observation in boys. Indeed, muscularity concerns significantly increase during adolescence [37,44] and muscle-gaining behaviors are shown to be more salient in popular boys [37]. Our findings raise the importance of recognizing not only overweightedness and obesity but also underweightedness as an issue regarding social relationships among adolescents.

WS and WP were significantly associated with having few confidants, after controlling for current depressive and anxiety symptoms. Having few confidants has been associated with subsequent development of mental health problems, including depressive disorder [45] and attempted self-harm [46]. The present results, taken together with these observations, suggest that adolescents with non-normal WS or WP might have increased risk of developing mental health problems associated with having few confidants, even if they do not currently have mental health problems. This seems to be in accordance with some previous studies, which find adverse effects of WS and WP on later developing mental health problems [47–52].

### Limitations

Our study has several limitations. First, this study used cross-sectional data, and the results might not explain causality. Second, BMI values were calculated using self-report height and weight, which may have affected estimations of BMI values in some groups; a recent meta-analysis observed that, at least for the screening of overweight and obesity in adolescents, self-report data is a valid alternative to directly measured height and weight [53]. Third, outcomes were measured using a single question. Single questions may be inadequate to accurately assess participants’ characteristics. For example, adolescents might have different reasons for their WP, including muscularity concerns and shape concerns about specific body parts. The present study also did not elaborate on which type of confiding relationships, such as family, friends, or teachers, underlay the number of confidants. Future studies on WS and WP should also take into account whether the number of friends or social network size may affect the number of confidants a person has, since a lower number of confidants may be a side effect of loss of friends due to experiencing social rejection, for example through bullying and weight-related marginalization [31,32]. Fourth, inadequate sample size for girls perceiving themselves to be *too* thin or *a bit* thin might have affected their associations with the number of confidants due to the lack of statistical power.

### Conclusions

The present study provides a novel perspective for understanding adolescents’ social and psychological problems in the light of body weight status and weight perception. As adolescents with non-normal WS and WP appear likely to have few confidants, more effort should be made to recognize and understand issues around social relationships in these adolescents and to give them more support in dealing with their challenges. Not only peers, but also family members and teachers should be made aware of the present findings and encouraged to look out for these adolescents.

## Supporting information

S1 Table Measurement of independent and dependent variables and covariates in the original language and English. (PDF)

S2 Table The distribution of weight status in boys and girls, stratified by school level, *n* (%). *n*, number of subjects.
(PDF)

S3 Table The distribution of weight perception in boys and girls, stratified by school level, *n* (%). *n*, number of subjects.
(PDF)

S4 Table The distribution of the number of confidants in boys and girls, stratified by school level, *n* (%). *n*, number of subjects.
(PDF)

## References

[1] Lerner R, Steinberg L, editors. Handbook of adolescent psychology volume 2: contextual influences on adolescent development 3rd ed Vol. 2 New York: John Wiley & Sons; 2009.

[2] Patton GC, Olsson CA, Skirbekk V, Saffery R, Wlodek ME, Azzopardi PS, et al Adolescence and the next generation. Nature. 2018;554(7693):458–66. 10.1038/nature25759 29469095

[3] Murray CJL. Quantifying the burden of disease: the technical basis for disability-adjusted life years. Bull World Health Organ. 1994;72(3):429–45. 10.1016/S0140-6736(96)07495-8 8062401 PMC2486718

[4] Bruce D, Ferguson BJ. Health for the world’s adolescents: a second chance in the second decade. J Adolesc Heal. 2015;56(1):3–6. 10.1016/j.jadohealth.2014.10.260 25530601

[5] Hall-Lande JA, Eisenberg ME, Christenson SL, Neumark-Sztainer D. Social isolation, psychological health, and protective factors in adolescence. Adolescence. 2007;42(166):265–86. 17849936

[6] Ponce M, Flores YN, Mudgal J, Huitrón G, Halley E, Gallegos-Carrillo K, et al The association between type of confidant and depressive symptomology in a sample of Mexican youth. Salud Ment. 2010;33(3):249–56.

[7] Endo K, Ando S, Shimodera S, Yamasaki S, Usami S, Okazaki Y, et al Preference for solitude, social isolation, suicidal ideation, and self-harm in adolescents. J Adolesc Heal. 2017;61(2):187–91. 10.1016/j.jadohealth.2017.02.018 28457686

[8] Brugha TS, Wing JK, Brewin CR, MacCarthy B, Lesage A. The relationship of social network deficits with deficits in social functioning in long-term psychiatric disorders. Soc Psychiatry Psychiatr Epidemiol. 1993;28(5):218–24. 10.1007/bf00788740 8284734

[9] Puhl RM, Latner JD. Stigma, obesity, and the health of the nation’s children. 2007;133(4):557–80. 10.1037/0033-2909.133.4.557 17592956

[10] Marathe A. Analysis of friendship network and its role in explaining obesity. ACM Intell Syst Technol. 2014;4(3):1–37. 10.1145/2483669.2483689 25328818 PMC4199209

[11] Crosnoe R, Frank K, Mueller AS. Gender, body size and social relations in American high schools. Soc Forces. 2007;86:1189.

[12] Baygi F, Kelishadi R, Qorbani M, Mohammadi F, Motlagh ME, Ardalan G, et al Association of some psychosocial factors with anthropometric measures in nationally representative sample of Iranian adolescents: the CASPIAN-III study. J Diabetes Metab Disord. 2016;15(1):1–11. 10.1186/S40200-016-0237-7 27252934 PMC4888475

[13] Ali MM, Amialchuk A, Rizzo JA. The influence of body weight on social network ties among adolescents. Econ Hum Biol. 2012;10(1):20–34. 10.1016/j.ehb.2011.10.001 22056235

[14] Schaefer MK, Blodgett EH. Eating Behaviors The connection of teasing by parents, siblings, and peers with girls’ body dissatisfaction and boys’ drive for muscularity: the role of social comparison as a mediator. Eat Behav. 2014;15(2014):599–608. 10.1016/j.eatbeh.2014.08.018 25218358

[15] Kjelgaard HH, Holstein BE, Due P, Brixval CS, Rasmussen M. Adolescent weight status: associations with structural and functional dimensions of social relations. J Adolesc Heal. 2017;60(4):460–8. 10.1016/j.jadohealth.2016.11.016 28110866

[16] Berge JM, Wall M, Larson N, Loth KA, Neumark-Sztainer D. Family functioning: associations with weight status, eating behaviors, and physical activity in adolescents. 2013;52(3):351–7. 10.1016/j.jadohealth.2012.07.006 23299010 PMC3580029

[17] ter Bogt TFM, van Dorsselaer SAFM, Monshouwer K, Verdurmen JEE, Engels RCME, Vollebergh WAM. Body Mass Index and body weight perception as risk factors for internalizing and externalizing problem behavior among adolescents. J Adolesc Heal. 2006;39(1):27–34. 10.1016/j.jadohealth.2005.09.007 16781958

[18] Mason A, Rantanen A, Kivim H, Koivisto A, Joronen K. Family factors and health behaviour of thin adolescent boys and girls. J Adv Nurs. 2016;73(1):177–89. 10.1111/jan.13096 27508504

[19] Sutin AR, Stephan Y, Wang L, Gao S, Wang P, Terracciano A. Personality traits and Body Mass Index in Asian populations. J Res Pers. 2015;58:137–42. 10.1016/j.jrp.2015.07.006 26327738 PMC4552249

[20] Allen MS, Walter EE. Personality and body image: a systematic review. Body Image. 2016;19:79–88. 10.1016/j.bodyim.2016.08.012 27636159

[21] Huang L, Tao FB, Wan YH, Xing C, Hao J, Su PY, et al Self-reported weight status rather than BMI may be closely related to psychopathological symptoms among Mainland Chinese adolescents. J Trop Pediatr. 2011;57(4):307–11. 10.1093/tropej/fmp097 19797398

[22] Griffiths S, Murray SB, Bentley C, Gratwick-Sarll K, Harrison C, Mond JM. Sex differences in quality of life impairment associated with body dissatisfaction in adolescents. J Adolesc Heal. 2017;61(1):77–82. 10.1016/j.jadohealth.2017.01.016 28389062

[23] Park E. Overestimation and underestimation: adolescents’ weight perception in comparison to BMI-based weight status and how it varies across socio-demographic factors. J Sch Heal. 2011;81(2):57–64. 10.1111/j.1746-1561.2010.00561.x 21223272

[24] Rayner KE, Schniering CA, Rapee RM, Taylor A, Hutchinson DM. Adolescent girls’ friendship networks, body dissatisfaction, and disordered eating: examining selection and socialization processes. J Abnorm Psychol. 2013;122(1):93–104. 10.1037/a0029304 22867115

[25] Cole TJ, Flegal KM, Nicholls D, Jackson AA. Body Mass Index cut offs to define thinness in children and adolescents: international survey. Br Med J. 2007;335(7612):194–7. 10.1136/bmj.39238.399444.55 17591624 PMC1934447

[26] Cole TJ, Bellizzi MC, Flegal KM, Dietz WH. Establishing a standard definition for child overweight and obesity worldwide: international survey. Br Med J. 2000;320(7244):1240–1240. 10.1136/bmj.320.7244.1240 10797032 PMC27365

[27] Joshy G, Korda RJ, Bauman A, Van Der Ploeg HP, Chey T, Banks E. Investigation of methodological factors potentially underlying the apparently paradoxical findings on body mass index and all-cause mortality. PLoS One. 2014;9(2):e88641 10.1371/journal.pone.0088641 24533128 PMC3923042

[28] Prospective Studies Collaboration. Body-Mass Index and cause-specific mortality in 900 000 adults: collaborative analyses of 57 prospective studies. Lancet. 2009;373(9669):1083–96. 10.1016/S0140-6736(09)60318-4 19299006 PMC2662372

[29] Berrington de Gonzalez A, Hartge P, Cerhan JR, Flint AJ, Hannan L, Macinnis RJ, et al Body-Mass Index and mortality among 1.46 million white adults. N Engl J Med. 2010;363:2211–9. 10.1056/NEJMoa1000367 21121834 PMC3066051

[30] Storch EA, Roth DA, Coles ME, Heimberg RG, Bravata EA, Moser J. The measurement and impact of childhood teasing in a sample of young adults. J Anxiety Disord. 2004;18(5):681–94. 10.1016/j.janxdis.2003.09.003 15275946

[31] Hayden-Wade HA, Stein RI, Ghaderi A, Saelens BE, Zabinski MF, Wilfley DE. Prevalence, characteristics, and correlates of teasing experiences among overweight children vs. non-overweight peers. Obes Res. 2005;13(8):1381–92. 10.1038/oby.2005.167 16129720

[32] Juvonen J, Lessard LM, Schacter HL, Suchilt L. Emotional implications of weight stigma across middle school: the role of weight-based peer discrimination. J Clin Child Adolesc Psychol. 2017;46(1):150–8. 10.1080/15374416.2016.1188703 27617887 PMC6105282

[33] van Harmelen AL, Gibson JL, St Clair MC, Owens M, Brodbeck J, Dunn V, et al Friendships and family support reduce subsequent depressive symptoms in at-risk adolescents. PLoS One. 2016;11(5):e0153715 10.1371/journal.pone.0153715 27144447 PMC4856353

[34] Tait RJ, Hulse GK, Robertson SI. A review of the validity of the General Health Questionnaire in adolescent populations. Aust N Z J Psychiatry. 2002;36(4):550–7. 10.1046/j.1440-1614.2002.01028.x 12169157

[35] Nakagawa Y, Daibo I. Manual of the Japanese version of the General Health Questionnaire Tokyo: Nihon Bunka Kagakusha; 1985.

[36] Honda S, Shibata Y, Nakane N. Screening of mental disorder with GHQ-12. J Heal Welf Stat. 2001;48(10):5–10.

[37] Rancourt D, Prinstein MJ. Peer status and victimization as possible reinforcements of adolescent girls’ and boys’ weight-related behaviors and cognitions. J Pediatr Psychol. 2010;35(4):354–67. 10.1093/jpepsy/jsp067 19667053 PMC2902838

[38] Marini M, Sriram N, Schnabel K, Maliszewski N, Devos T, Ekehammar B, et al Overweight people have low levels of implicit weight bias, but overweight nations have high levels of implicit weight bias. PLoS One. 2013;8(12):1–9. 10.1371/journal.pone.0083543 24358291 PMC3866190

[39] Puhl RM, Heuer CA. The stigma of obesity: a review and update. Obesity. 2009;17(5):941–64. 10.1038/oby.2008.636 19165161

[40] Simpkins SD, Schaefer DR, Price CD, Vest AE. Adolescent friendships, BMI, and physical activity: untangling selection and influence through longitudinal social network analysis. J Res Adolesc. 2013;23(3):1–21. 10.1111/j.1532-7795.2012.00836.x 24222971 PMC3821778

[41] Bully P, Elosua P. Changes in body dissatisfaction relative to gender and age: the modulating character of BMI. Span J Psychol. 2011;14(1):313–22. 10.5209/rev_sjop.2011.v14.n1.28 21568188

[42] McCabe MP, Ricciardelli LA. Body image dissatisfaction among males across the lifespan: a review of past literature. J Psychosom. 2004;56(6):675–85. 10.1016/S0022-3999(03)00129-6 15193964

[43] Cohane GH, Pope HG. Body image in boys: a review of the literature. Int J Eat Disord. 2001;29(4):373–9. 10.1002/eat.1033 11285574

[44] Hoffmann S, Warschburger P. Weight, shape, and muscularity concerns in male and female adolescents: predictors of change and influences on eating concern. Int J Eat Disord. 2017;50(2):139–47. 10.1002/eat.22635 27739586

[45] Brugha TS, Weich S, Singleton N, Lewis G, Bebbington PE, Jenkins R, et al Primary group size, social support, gender and future mental health status in a prospective study of people living in private households throughout Great Britain. Psychol Med. 2005;35(5):705–14. 10.1017/s0033291704003903 15918347

[46] Leverich GS, Altshuler LL, Frye MA, Suppes T, E KP, McElroy SL, et al Factors associated with suicide attempts in 648 patients with bipolar disorder in the Stanley Foundation Bipolar Network. J Clin Psychiatry. 2003;64(5):506–15. 10.4088/jcp.v64n0503 12755652

[47] Sutaria S, Devakumar D, Yasuda SS, Das S, Saxena S. Is obesity associated with depression in children? Systematic review and meta-analysis. Arch Dis Child. 2018;104(1):64–74. 10.1136/archdischild-2017-314608 29959128

[48] Ikeda M, Tanaka S, Saito T, Ozaki N, Kamatani Y, Iwata N. Re-evaluating classical body type theories: genetic correlation between psychiatric disorders and Body Mass Index. Psychol Med. 2018;48(10):1745–8. 10.1017/S0033291718000685 29651975 PMC6088781

[49] Sormunen E, Saarinen MM, Salokangas RKR, Hutri-kähönen N, Viikari JSA, Raitakari OT, et al Body Mass Index trajectories in childhood and adolescence—risk for non-affective psychosis. Schizophr Res. 2018;S0920-9964(18):30627–33. 10.1016/j.schres.2018.10.025 30482644

[50] Al Mamun A, Cramb S, McDermott BM, O’Callaghan M, Najman JM, Williams GM. Adolescents’ perceived weight associated with depression in young adulthood: a longitudinal study. Obesity. 2007;15(12):3097–105. 10.1038/oby.2007.369 18198320

[51] Whitehead RD, Cosma A, Cecil J, Currie C, Currie D, Neville F, et al Trends in the perceived body size of adolescent males and females in Scotland, 1990–2014: changing associations with mental well-being. Int J Public Health. 2018;63(1):69–80. 10.1007/s00038-017-0997-y 28668973 PMC5766710

[52] Xie B, Reynolds K, Palmer PH, Gallaher P, Anderson JC, Wu Q, et al Longitudinal analysis of weight perception and psychological factors in Chinese adolescents. Am J Heal Behav. 2011;35(1):92–104. 10.5993/ajhb.35.1.9 20950162 PMC2957668

[53] He J, Cai Z, Fan X. Accuracy of using self-reported data to screen children and adolescents for overweight and obesity status: a diagnostic meta-analysis. Obes Res Clin Pract. 2017;11(3):257–67. 10.1016/j.orcp.2017.03.004 28389205

