# Supplementary material for: The association of weight status and weight perception with number of confidants in adolescents
Source: PLoS One. 2019 Dec 4;14(12):e0225908. doi: 10.1371/journal.pone.0225908 (PMC6892562; doi:10.1371/journal.pone.0225908)
Supplement: S1 Table — (PDF) [file pone.0225908.s001.pdf]

Variable	Item	Response options
**Weight status**	現在のあなたの身長と体重を教えてください。<br>Please fill in your height and weight.	_________ cm, _________ kg
**Weight perception**	現在の体重についてどう感じていますか？<br>What do you think of your current body weight?	太りすぎている，少し太っている，ちょうどよい，少しやせている，やせすぎている<br>Too fat, A bit fat, Good, A bit thin, Too thin
**Number of confidants**	なやみごとや心配事を相談できる人は何人いますか？<br>How many people are there for you to confide in about your problems/concerns?	いない, 1 人, 2 人, 3 人, 4 人以上<br>None, 1, 2, 3, 4 or more
**Experience of being bullied**	この１年以内に、いじめられたことはありましたか？<br>Have you been bullied within the past year?	なかった，あった<br>No, Yes
