# Supplementary material for: The association of weight status and weight perception with number of confidants in adolescents
Source: PLoS One. 2019 Dec 4;14(12):e0225908. doi: 10.1371/journal.pone.0225908 (PMC6892562; doi:10.1371/journal.pone.0225908)
Supplement: S2 Table — n, number of subjects. (PDF) [file pone.0225908.s002.pdf]

	Underweight	Low-normal weight	Mid-normal weight	High-normal weight	Overweight
	**Boys (N = 8,108)**				
**Junior high**	337 (8.8)	748 (19.5)	1,657 (43.2)	684 (17.8)	406 (10.6)
**Senior high**	379 (8.9)	862 (20.2)	1,745 (40.8)	772 (18.1)	518 (12.1)
	**Girls (N = 7,171)**				
**Junior high**	401 (12.0)	770 (23.2)	1,379 (41.6)	539 (16.3)	227 (6.8)
**Senior high**	521 (13.5)	995 (25.8)	1,503 (39.0)	592 (15.4)	244 (6.3)
