# Supplementary material for: The association of weight status and weight perception with number of confidants in adolescents
Source: PLoS One. 2019 Dec 4;14(12):e0225908. doi: 10.1371/journal.pone.0225908 (PMC6892562; doi:10.1371/journal.pone.0225908)
Supplement: S3 Table — n, number of subjects. (PDF) [file pone.0225908.s003.pdf]

	***Too* thin**	***A bit* thin**	**Good**	***A bit* fat**	***Too* fat**
	**Boys (*N* = 8,108)**				
**Junior high**	206 (5.4)	737 (19.2)	1,774 (46.3)	854 (22.3)	261 (6.8)
**Senior high**	383 (9.0)	873 (20.4)	1,517 (35.5)	1,056 (24.7)	447 (10.5)
	**Girls (*N* = 7,171)**				
**Junior high**	44 (1.3)	202 (6.1)	998 (30.1)	1,459 (44.0)	613 (18.5)
**Senior high**	43 (1.1)	126 (3.3)	734 (19.0)	1,869 (48.5)	1,083 (28.1)
