# Supplementary material for: The association of weight status and weight perception with number of confidants in adolescents
Source: PLoS One. 2019 Dec 4;14(12):e0225908. doi: 10.1371/journal.pone.0225908 (PMC6892562; doi:10.1371/journal.pone.0225908)
Supplement: S4 Table — n, number of subjects. (PDF) [file pone.0225908.s004.pdf]

	**0–3**	**4 or more**
	**Boys (*N* = 8,108)**	
**Junior high**	2,075 (54.1)	1,757 (45.9)
**Senior high**	2,592 (60.6)	1,684 (39.4)
	**Girls (*N* = 7,171)**	
**Junior high**	1,866 (56.2)	1,450 (43.7)
**Senior high**	2,327 (60.3)	1,528 (39.6)
