# Supplementary material for: The association of weight status and weight perception with number of confidants in adolescents
Source: PLoS One. 2019 Dec 4;14(12):e0225908. doi: 10.1371/journal.pone.0225908 (PMC6892562; doi:10.1371/journal.pone.0225908)
Supplement: Attachment — Submitted filename: Response to Reviewers.docx [file pone.0225908.s005.docx]

Response to Reviewers:

Thank you all very much for your constructive comments. We have carefully considered all the points addressed and the feedback and have accordingly made substantial changes to the manuscript. In this response letter, original comments by the Academic Editor and Reviewers are indicated in **bold**. Our responses are written in regular text. Locations of each part that was revised are indicated in red. Manuscript text is indented and indicated in “*quotations and italics*”, and changes to the text are indicated in *blue*. In the manuscript file, changes to the text are also indicated in blue.

**Response to comments by Academic Editor (AE)**

**AE – Comment 1:**

**When submitting your revision, we need you to address these additional requirements.**

**Please ensure that your manuscript meets PLOS ONE's style requirements, including those for file naming. The PLOS ONE style templates can be found at**
[**http://www.journals.plos.org/plosone/s/file?id=wjVg/PLOSOne_formatting_sample_main_body.pdf**](http://www.journals.plos.org/plosone/s/file?id=wjVg/PLOSOne_formatting_sample_main_body.pdf) **and** [**http://www.journals.plos.org/plosone/s/file?id=ba62/PLOSOne_formatting_sample_title_authors_affiliations.pdf**](http://www.journals.plos.org/plosone/s/file?id=ba62/PLOSOne_formatting_sample_title_authors_affiliations.pdf)

**Response:**

We apologize for not meeting the journal requirements, which should have been done before the first submission. Corrections have been made in accordance with the journal style guidelines and templates.

**AE – Comment 2:**

**You indicated that you had ethical approval for your study. In your Methods section, please ensure you have also stated whether you obtained consent from parents or guardians of the minors included in the study or whether the research ethics committee or IRB specifically waived the need for their consent.**

**Response:**

We apologize for the inadequate statement of the ethical approval for our study. We offered the possibility of opting-out rather than obtaining consent. Parents and guardians received a letter that asked them to notify the school if they withheld consent for their child’s participation in the present research. This was approved by the ethics committees. To clarify the above, we have added a sentence to the text in the Materials and methods section as follows.

Populations and procedures sub-section, page 6, lines 105–107:

“*The principals then consulted with the* *teachers. The parents and guardians received a letter from the principal investigators that asked them to notify the school if they withheld consent for their child’s participation in the present research.*”

**AE – Comment 3:**

**Please include additional information regarding the survey or questionnaire used in the study and ensure that you have provided sufficient details that others could replicate the analyses. For instance, if you developed a questionnaire as part of this study and it is not under a copyright more restrictive than CC-BY, please include a copy, in both the original language and English, as Supporting Information.**

**Response**

Thank you for the request. A questionnaire was indeed developed for our research, and we selected some of the items from the questionnaire to conduct the current analyses. We have now made a Supporting Information section and provided S1 Table, which includes the questionnaire items, in the original language and English, that were used in the analyses. All the coauthors agreed to providing the information. Subsequently, the following paragraph was added to the text in the Materials and methods section.

Measurement sub-section, page 7, lines 128–131:

“*The questionnaire items and response options used in the present study are shown in S1 Table, including the measurement of independent and dependent variables and covariates in the original language and English.* *Details of the coding for each variable are described in the following sub-sections.*”

The following supplementary table was included to Supporting Information:

**S1 Table. Measurement of Independent and Dependent Variables and Covariates in the Original Language and English.**

| Variable | Item | Response options |
| --- | --- | --- |
| Weight status | 現在のあなたの身長と体重を教えてください。  Please fill in your height and weight. | _________ cm, _________ kg |
| Weight perception | 現在の体重についてどう感じていますか？  What do you think of your current body weight? | 太りすぎている, 少し太っている, ちょうどよい, 少しやせている, やせすぎている  Too fat, A bit fat, Good, A bit thin, Too thin |
| Number of confidants | なやみごとや心配事を相談できる人は何人いますか？  How many people are there for you to confide in about your problems/concerns? | いない, 1人, 2人, 3人, 4人以上  None, 1, 2, 3, 4 or more |
| Experience of being bullied | この１年以内に、いじめられたことはありましたか？  Have you been bullied within the past year? | なかった, あった  No, Yes |

**Response to the comments by the Reviewer 1 (R1)**

**R1 – Comment 1:**
**Authors presented BMI cut-off by WHO, however these values are applicable only for adult population. For children growth reference charts must be applied – e.g. WHO recommendations for children under 18 years old. If in Japan national charts exist, it is an even better situation because of a better fitting for the Japanese adolescent population. Therefore, the national growth reference charts should be applied.**

**Response:**

We apologize for the misleading explanations. We gave careful consideration when selecting BMI cut-offs for the present analyses. The present study applied the BMI cut-off values for children and adolescents under 18 (i.e. Cole et al. 2007, Body Mass Index cut-offs to define thinness in children and adolescents: international survey, Reference 25; Cole et al. 2000, Establishing a standard definition for child overweight and obesity worldwide: international survey, Reference 26). These references are widely used in body weight research to determine weight status in adolescents. We did not apply the Japanese national growth reference charts because, instead of BMI, they use an obesity index which is less widely used. Hence, we decided to apply internationally defined BMI cut-offs in our analyses. To clarify the above issue, a few expressions have been added to the text in the Materials and methods section as follows.

Weight status and weight perception sub-section, page 8, lines 133–137:

“*BMI values were initially split into four WS categories (underweight, normal weight, overweight, and obese), using BMI cut-off values that are equivalent to adult BMI values of 18.5, 25.0, and 30.0, based on age- and sex-specific cut-off values for underweightedness [25] and overweightedness and obesity [26] in adolescents.*”

For the actual cut-off values used for categorizing underweightedness (i.e. adult BMI value of 18.5) and overweightedness (i.e. adult BMI value of 25.0 and 30.0) in adolescents, please refer to the tables drawn from Cole et al. (2000 and 2007) attached below:

Reference 25: *Cole TJ, Flegal KM, Nicholls D, Jackson AA. Body Mass Index cut offs to define thinness in children and adolescents: international survey. Br Med J. 2007;335(7612):194–7. doi: 10.1136/bmj.39238.399444.55*

Reference 26: *Cole TJ, Bellizzi MC, Flegal KM, Dietz WH. Establishing a standard definition for child overweight and obesity worldwide: international survey. Br Med J. 2000;320(7244):1240–1240. doi: 10.1136/bmj.320.7244.1240*

**R1 – Comment 2:**
**Moreover, Authors should present the number of ethics committee's approval.**

**Response:**

Thank you for the request. We have added the numbers of the ethics committee’s approval to the text in the Materials and methods section, as follows.

Populations and procedures sub-section, page 7, lines 123–126:

“*The data collection was approved by the ethics committees of the Tokyo Metropolitan Institute of Psychiatry (approval number: 20-9), the Mie University School of Medicine (approval number: 603), and Kochi Medical School at Kochi University (approval number: 20-57).*”

**R1 – Comment 3:**
**Authors also should complete the information about the statistical tests that have been applied. How did Authors verify the normality of data distribution? What tests did they used?**

**Response:**

We apologize for the confusing expression and wrong choice of words. We believe that R1 – Comment 3 is based on line 137, the phrase underlined in the following: “*Data were distributed normally; the majority of subjects fell in the normal weight category.*” It was our mistake in using the wrong expression; we did not mean to say that the distribution of data was a ‘normal distribution’. Our intention was rather to emphasize the other half of the sentence (i.e. “*the majority of subjects fell in the normal weight category*”) in order to show the reason for further dividing the normal weight group into smaller categories. We have removed the phrase “*Data were distributed normally;*” from the text. The change has been made in the Materials and methods section as follows:

Weight status and weight perception sub-section, page 8, line 137:

“*BMI values were initially split into four WS categories (underweight, normal weight, overweight, and obese), using BMI cut-off values that are equivalent to adult BMI values of 18.5, 25.0, and 30.0, based on age- and sex-specific cut-off values for underweightedness [25] and overweightedness and obesity [26] in adolescents. ~~Data were distributed normally;~~ The majority of subjects fell in the normal weight category.*”

**R1 – Comment 4:
In my opinion, anthropometric measurements should be made. Especially, taking into account that another authors indicate that more than half middle- and high-school students, did not correctly perceive their own body weight (Yan et al. 2018. Body Weight Misperception and Its Association with Unhealthy Eating Behaviors among Adolescents in China. IJERPH.). In such situation, the own perception of body mass cannot be perceived as a valid measurement.**

**Response:**

Thank you for the feedback. As pointed out, the present study used self-report body height and weight for the approximate anthropometric measurements of body mass. This is considered as a limitation of the present study, although a recent meta-analysis suggested that self-reported data could be a valid alternative to directly measured height and weight, at least in the screening of overweight and obesity in adolescents. We discussed about this in the Limitations sub-section, page 18, lines 303–306, as follows (please also note that we have corrected some sentence expressions):

“*Second, BMI values were calculated using self-report height and weight, which may have affected estimations of BMI values in some groups**;* *a recent meta-analysis observed that, at least for the screening of overweight and obesity in adolescents, self-report data is a valid alternative to directly measured height and weight [51].*”

(Yan et al. 2018 compared body weight perception (WP) with BMI calculated using self-reported height and weight, and found large discrepancies between these variables, but not between self-reported and objectively measured height and weight; height and weight were not objectively measured in their study.)

**Responses to the comments by the Reviewer 2 (R2)**

**R2 – Comment 1:**

**Please include explanation on why WS and WP were both used in the study in the Introduction Section.**

**Response:**

Thank you for the feedback. The reasons for using WS, as stated in the Introduction section, lines 57–78, include weight stigmatization in the surroundings, small social network size, relationship difficulties with family and friends in those who are overweight, and difficulties in social behavior and introversion in those who are underweight. In addition to WS, WP was used in our study because WP is often discordant with WS (Park 2011*). Furthermore, the effect of WP may not be in accordance with the effect of the corresponding WS. For example, a perception of being fat or thin might not affect social relationships as negatively as overweight or underweight might, given that girls may be more likely to become friends with other girls who have similar weight/shape concerns (Rayner 2013, Reference 24). We addressed this in the Discussion section, lines 273–275, and now we have also added this information to the introduction. To reflect the above details, one study has been added to the reference list, numbered Reference 23. In addition, an explanation of the use of WP in addition to WS has been added to the text as follows.

*Addition to the Reference list, page 23, lines 399–401:

23. *Park E. Overestimation and underestimation: adolescents’ weight perception in comparison to BMI-based weight status and how it varies across socio-demographic factors. J Sch Health. 2011;81(2):57-64. doi:10.1111/j.1746-1561.2010.00561.x.*

Introduction section, page 5, lines 82–90:

“*Previous studies have also observed that adolescents who perceive themselves as either being thin or fat report more social adaptation problems (e.g. problems becoming accustomed to school life) [17,21] and impairment in social functioning (e.g. trouble getting along with peers) [22].* *In addition, a study observed that WP was discordant with WS in a quarter of adolescents [23].* *Furthermore, the effect of WP may not be in accordance with the effect of the corresponding WS. For example, a perception of being either thin or fat might not affect social relationships as negatively as actually being underweight or overweight might, given that girls may be more likely to become friends with other girls who have similar weight concerns [24].*”

**R2 – Comment 2:
Please state the sampling method used in the Methodology Section.**

**Response:**

Thank you for addressing this point. An important expression was missing in the original paragraph – to explain that the school principals of ‘all’ public junior and senior high schools in Kochi prefecture and public junior high schools in Tsu City, Mie prefecture, were approached for the data collection. We believe that describing this in the Population and procedures sub-section in Materials and methods section will be a sufficient addition to the main text in place of stating the sampling method. The sentences have been added to the text as follows.

Population and procedures sub-section, page 6, lines 101–109:

“*The principal investigators approached the school principals of all public junior and senior high schools in Kochi prefecture (population: 780,000), and all public junior high schools in Tsu City (population: 290,000) in Mie prefecture. School principals were told that participation in the survey was voluntary. The principals then consulted with the teachers. The parents and guardians received a letter that asked them to notify the school if they withheld consent for their child’s participation in the present research. Among the 138 public junior and 36 public senior high schools, 45 junior high and 28 senior high schools participated in this study.*”

**R2 – Comment 3:**

**Please justify the division of weight category into low-normal weight, mid-normal weight, and high-normal weight (Line 132).**

**Response:**

Thank you for the comment. BMI cut-offs by Cole et al. (2000 and 2007) only offer a single category for normal weight, which resulted in approximately 80% of the sample falling into the normal weight group. In such a situation, the effects of being underweight and overweight on the number of confidants might be underestimated due to including those who are close to the borderline between case and non-case categories in the reference group (i.e. normal weight group). For example, adolescents with higher BMI, but not necessarily categorized as being overweight, have been associated with lower levels of likability and popularity (Rancourt 2010, Reference 35). A previous study also showed that amongst female adolescents, the ideal BMI category is characterized by low normal weight, and that amongst male adolescents by high normal weight (Bully 2011, Reference 39). These observations may suggest that being closer to either end of the normal weight category might to some extent have adverse effects on social relationships.

Regarding the cut-off values, 20.0 and 22.5 are used in keeping with previous large studies with 0.25–1.5 million participants (Joshy 2014, Reference 27; Prospective Studies Collaboration 2009*; Berrington de Gonzalez 2010*). Joshy 2014 was already cited in the original text. The other two studies were added to the reference list, numbered Reference 28 and Reference 29. Consequently, a few changes have been made to the text in the Materials and methods section as follows.

Weight status and weight perception sub-section, page 8, lines 138–140:

“*Thus, we further divided the normal weight category into low-normal weight, mid-normal weight, and high-normal weight using cut-off points which are comparable to the BMI values of 20.0 and 22.5 for adults [27–29].*”

*Additions to the Reference list, pages 24–25, lines 416–421:

28. *Prospective Studies Collaboration. Body-Mass Index and cause-specific mortality in 900 000 adults: collaborative analyses of 57 prospective studies. Lancet. 2009;373:1083-96. doi: 10.1016/S0140-6736(09)60318-4*
29. *Berrington de Gonzalez A, Hartge P, Cerhan JR, Flint AJ, Hannan L, Maclnnis RJ, et al. Body-Mass Index and mortality among 1.46 million white adults. N Engl J Med. 2010;363:2211-9. doi: 10.1056/NEJMoa1000367*

**R2 – Comment 4:
Please state the Operational Definition of 'WS, WP and Confidants' in the Methodology.**

**Response:**

Thank you for the feedback. In addition to including S1 Table which shows questionnaire items of independent and dependent variables and covariates in the original language and English, we have also incorporated a few more details in each sub-section in the Materials and methods section so that the coding for each variable is defined clearly.

Weight status and weight perception sub-section, page 8, lines 140–146:

“*Finally, the number of divisions for WS was reduced from 6 to 5 groups by merging the obesity group into the overweight group, since the number of obese subjects was small (n = 224 in boys; n = 67 in girls). In the end, the five WS categories were defined, with cut-off values adjusted for age and sex corresponding to the following equivalent BMIs in adults:* *Underweight: BMI < 18.5; Low-normal weight: 18.5 ≤ BMI < 20.0; Mid-normal weight: 20.0 ≤ BMI < 22.5; High-normal weight: 22.5 ≤ BMI < 25.0; Overweight: BMI ≥ 25.0*.”

Weight status and weight perception sub-section, pages 8–9, lines 147–151:

“*WP was measured using the following one-item question: "What do you think of your current body weight?" The students were given a five-point Likert scale with responses defined as follows: 1. “Too fat”; 2. “A bit fat”; 3. “Good”; 4. “A bit thin”; and 5. “Too thin”. The students responded to the question by circling one of the given choices. In the analyses, each answer was considered as one category.*”

Number of confidants sub-section, page 9, lines 153–159:

“*The number of confidants was assessed with the following question, “How many people are there for you to confide in about your problems/concerns?”, with five possible answers: 1. “None”; 2. “One”; 3. “Two”; 4. “Three”; 5. “Four or more”. The students responded to the question by circling one of the given choices. Adolescents with 3 or fewer confidants were considered as having few confidants. A dichotomous variable was then created by combining answers of 1 to 4 into the “0*–*3 (Few) confidants” group, and putting answers of 5 in the “≥ 4 (More) confidants” group.*”

**R2 – Comment 5:
Since this study involved adolescents grade 7-12 (junior and high schools), was there any difference in terms of WS, WP and confidants numbers between age groups or school levels? Please elaborate more on age factor in the discussion.**

**Response:**
Thank you for the comment. We have provided S2–4 Tables to show the distribution of WS, WP and the number of confidants, stratified by school level. (In Japan, students in any given grade at school are uniformly of the same age.) As shown in the Tables, the shapes of the distributions were generally the same across school levels (i.e. the same category always showed the largest proportion for boys and girls within each variable), although the prevalence for WP in girls was slightly different between junior and senior high school students. We therefore did not stratify the analyses by school level. Accordingly, we added a few sentences to the text in the Results section as follows.

Descriptive statistics sub-section, pages 11–12, lines 187–192:

“*WP for boys had a normal distribution, while the distribution of girls was skewed toward the perception of being fat.* *The distributions of WS, WP, and the number of confidants did not differ substantially across school level (**Junior High / Senior High); in both boys and girls, the same category always showed the largest proportion within each variable. The distributions of* *WS, WP, and the number of confidants, stratified by school level are shown in S2*–*4 Tables.*”

The following supplementary tables were included to Supporting Information:

**S2 Table. The Distribution of Weight Status in Boys and Girls, Stratified by School Level, *n* (%).**

|  | **Underweight** | **Low-normal weight** | **Mid-normal weight** | **High-normal weight** | **Overweight** |
| --- | --- | --- | --- | --- | --- |
|  | **Boys (*N* = 8,108)** | | | | |
| **Junior high** | 337 (8.8) | 748 (19.5) | 1,657 (43.2) | 684 (17.8) | 406 (10.6) |
| **Senior high** | 379 (8.9) | 862 (20.2) | 1,745 (40.8) | 772 (18.1) | 518 (12.1) |
|  | **Girls (*N* = 7,171)** | | | | |
| **Junior high** | 401 (12.0) | 770 (23.2) | 1,379 (41.6) | 539 (16.3) | 227 (6.8) |
| **Senior high** | 521 (13.5) | 995 (25.8) | 1,503 (39.0) | 592 (15.4) | 244 (6.3) |

*n*, number of subjects.

**S3 Table. The Distribution of Weight Perception in Boys and Girls, Stratified by School Level, *n* (%).**

|  | ***Too* thin** | ***A bit* thin** | **Good** | ***A bit* fat** | ***Too* fat** |
| --- | --- | --- | --- | --- | --- |
|  | **Boys (*N* = 8,108)** | | | | |
| **Junior high** | 206 (5.4) | 737 (19.2) | 1,774 (46.3) | 854 (22.3) | 261 (6.8) |
| **Senior high** | 383 (9.0) | 873 (20.4) | 1,517 (35.5) | 1,056 (24.7) | 447 (10.5) |
|  | **Girls (*N* = 7,171)** | | | | |
| **Junior high** | 44 (1.3) | 202 (6.1) | 998 (30.1) | 1,459 (44.0) | 613 (18.5) |
| **Senior high** | 43 (1.1) | 126 (3.3) | 734 (19.0) | 1,869 (48.5) | 1,083 (28.1) |

*n*, number of subjects.

**S4 Table. The Distribution of the Number of Confidants in Boys and Girls, Stratified by School Level, *n* (%).**

|  | **0**–**3** | **4 or more** |
| --- | --- | --- |
|  | **Boys (*N* = 8,108)** | |
| **Junior high** | 2,075 (54.1) | 1,757 (45.9) |
| **Senior high** | 2,592 (60.6) | 1,684 (39.4) |
|  | **Girls (*N* = 7,171)** | |
| **Junior high** | 1,866 (56.2) | 1,450 (43.7) |
| **Senior high** | 2,327 (60.3) | 1,528 (39.6) |

*n,* number of subjects.

**Responses to the comments by the Reviewer 3 (R3)**

**R3 – Comment 1:
Abstract: I suggest including design of study in abstract.**

**Response:**

Thank you for the suggestion. We have added ‘cross-sectional’ in the Abstract section, page 2, line 32. The change has been made to the text as follows:

“*This preliminary cross-sectional study examined the effects of weight status and weight perception on the number of confidants in adolescents.*”

**R3 – Comment 2:
Introduction: 1. The introduction is adequate, however I suggest excluding this sentence “Analyses of the associations are stratified by sex and further adjusted for age, experience of being bullied, and depressive and anxiety symptoms.”**

**Response:**
Thank you for the suggestion. We agree that the sentence mentioned in the comment does not belong to the Introduction section but rather overlaps with the Materials and methods section. As suggested, we have removed the sentence from the text.

**R3 – Comment 3:**

**Methods: 1. Was there sample calculation? Please make it clear.**

**Response:**

Thank you for the advice. We have calculated the required sample size following the advice. It was calculated, for the binary logistic regression, as 2,548 references versus 849 cases, when *α* = .05 and *β* = 0.1, with the assumptions of the proportion of adolescents who have few confidants in the reference group = 0.4, OR = 1.3, and the ratio of the references versus cases = 3:1. When the ratio of the references versus cases is assumed = 1:1, the sample size required is calculated as 1,276 references versus 1,276 cases. The actual sample size, summarized in Table 1, seems to be close to the calculated sample size, except for the groups of “underweight” boys and “overweight (plus obese)” girls among WS categories (please note that “obese” and “overweight” were merged into the “overweight” category in the analysis) and the groups of boys and girls perceiving themselves to be “*too* thin”, boys perceiving themselves to be “*too* fat”, and girls perceiving themselves to be “*a bit* thin” among WP categories. Among these groups, no significant associations were observed between WS/WP and the number of confidants in girls perceiving themselves to be “*a bit* thin” and “*too* thin”. (The groups that had inadequate sample sizes according to the sample calculation are highlighted in yellow in Table 1 attached below.)

**Table 1. Weight Status, Weight Perception, the Number of Confidants, Experience of Being Bullied, and the General Health Questionnaire (GHQ)-12 Scores in Boys and Girls.**

|  | **Boys** | **Girls** |
| --- | --- | --- |
| ***N*** | 8,108 | 7,171 |
| **Weight status, *n* (%)** | | |
| Underweight | 716 (8.8) | 922 (12.9) |
| Low-normal weight | 1,610 (19.9) | 1,765 (24.6) |
| Mid-normal weight | 3,402 (42.0) | 2,882 (40.2) |
| High-normal weight | 1,456 (18.0) | 1,131 (15.8) |
| Overweight | 699 (8.6) | 404 (5.6) |
| Obese | 225 (2.8) | 67 (0.9) |
| **Weight perception, *n* (%)** | | |
| *Too* thin | 589 (7.3) | 87 (1.2) |
| *A bit* thin | 1,610 (19.9) | 328 (4.6) |
| Good | 3,291 (40.6) | 1,732 (24.2) |
| *A bit* fat | 1,910 (23.6) | 3,328 (46.4) |
| *Too* fat | 708 (8.7) | 1,696 (23.7) |

We have added this to the Results section and Limitations sub-section as follows.

Binary logistic regression sub-section, pages 14–15, lines 226–238:

“*According to the sample calculation, the required sample size for the present binary logistic regression was shown to be 2,548 references versus 849 cases, when α = .05 and β = 0.1, with the assumptions of the proportion of adolescents who have few confidants in the reference group = 0.4, OR = 1.3, and the ratio of the references versus cases = 3:1. When the ratio of the references versus cases is assumed = 1:1, the sample size required is calculated as 1,276 references versus 1,276 cases**.* *The actual sample size, summarized in Table 1, seems to be close to the calculated sample size, except for the groups of “underweight” boys and “overweight (plus obese)” girls among WS categories (note that “obese” and “overweight” were merged into the “overweight” category in the analysis), and the groups of boys and girls perceiving themselves to be “too thin”, girls perceiving themselves to be “a bit thin”, and boys perceiving themselves to be “too fat” among WP categories. Among these groups, no significant associations between WS/WP and the number of confidants were observed in girls perceiving themselves to be “a bit thin” and “too thin”.*”

Limitations sub-section, page 19, lines 315–317:

“*Fourth, inadequate sample size for girls perceiving themselves to be too thin or a bit thin might have affected their associations with the number of confidants due to the lack of statistical power.*”

**R3 – Comment 4:
Methods: 2. Why the authors decided to use Cole et al (2000 and 2007) as the reference of BMI cut-off? These BMI cut-off values are adequate to evaluate anthropometric status in children and adolescents, but have as limitations to use the same cut-offs for men and women.**

**Response:**

Thank you for the comment. We gave careful consideration when selecting BMI cut-offs for the present analysis. The adolescent BMI cut-off values drawn from Cole et al. (2000 and 2007) do take into account the gender differences – in their tables, the authors present age-specific cut-offs for boys and girls respectively. Please refer to the tables drawn from Cole et al. (2000 and 2007) attached below:

Reference 25: *Cole TJ, Flegal KM, Nicholls D, Jackson AA. Body Mass Index cut offs to define thinness in children and adolescents: international survey. Br Med J. 2007;335(7612):194–7. doi: 10.1136/bmj.39238.399444.55*

Reference 26: *Cole TJ, Bellizzi MC, Flegal KM, Dietz WH. Establishing a standard definition for child overweight and obesity worldwide: international survey. Br Med J. 2000;320(7244):1240–1240. doi: 10.1136/bmj.320.7244.1240*

**R3 – Comment 5:
Methods: 3. Were the tools validated for adolescents? Could you indicate the alpha data? Please complete this.**

**Response:**
Thank you for the comment. If ‘the alpha’ in the comment reflects Cronbach’s alpha, then we believe that it is not necessary for the present study to indicate the alpha because we did not use any tools that require scoring. If ‘the tools’ reflects the BMI cut-offs by Cole et al. (2000 and 2007), these cut-offs are widely used references in body weight research to determine WS in adolescents.

**R3 – Comment 6:
Results: 1. I suggest avoiding the use of single-sentence paragraphs, for instance “The largest proportion of students (boys: 41.7%, girls: 40.1%) fell into the mid-normal weight group (Table 1).”**

**Response:**

We apologize for the confusion and wrong arrangement of the tables. Lines 185–186 “*The largest proportion of students (boys: 41.7%, girls: 40.1%) fell into the mid-normal weight group (Table 1).*” and line 206 “*The results for the binary logistic regression analyses are summarized in Table 3.*” are not supposed to be single-sentence paragraphs. The Results section rather consisted of two paragraphs; one paragraph for the Descriptive Statistics sub-section, pages 11–12, lines 185–196, and the other for the Binary logistic regression sub-section, pages 14–15, lines 206–238. Therefore, Tables 1 and 2 go after the Descriptive Statistics paragraph and Table 3 after the Binary logistic regression paragraph. No single-sentence paragraphs are left in the manuscript after making these corrections.

**R3 – Comment 7:
Results: 2. I suggest writing this sentence “The prevalence of being underweight 177 was 8.7% in boys and 12.7% in girls, while being overweight was 14.2% in boys and 7.5% in girls. WP for boys had a normal distribution, while the distribution of girls was skewed toward the perception of being fat.” before table 1.**

**Response:**
Thank you for the advice. Corrections reflecting R3 – Comment 6 cover the suggestion in the present comment.

**R3 – Comment 8:
Discussion: 1. In the first paragraph include for boys the association found between to be a bit fat and to have few confidants among adolescents.**

**Response:**

Thank you for the request. We agree that the association between being a bit fat and having few confidants among boys was missing in the text. A few changes have been made to the original text as follows.

Discussion section, page 15, lines 246–247:

“*Girls who were high-normal weight were also more likely to have few confidants, as were boys who were underweight or perceived themselves to be too thin* *or a bit fat.*”

**R3 – Comment 9:
Discussion: 2. I suggest improving the discussion for the result presented on pages 16 and 17, lines 231 to 236, about the association found between feeling a bit fat and to have few confidants among boys.**

**Response:**
Thank you for the comment. Following the advice, we have added text as follows.

Discussion section, page 17, lines 275–278:

“*In the present study, more than half of the girls reported perceiving themselves to be a bit fat. In boys, the proportion was half of that in girls.* *This may suggest that the perception of being a bit fat may actually be “normal” in girls, mitigating any negative effects on the number of confidants,* *but not in boys.*”

**R3 – Comment 10:**

**Conclusion: Conclusions are adequate.**

**Response:**

Thank you for the feedback.

**Additional corrections**

We discovered some small errors in Tables 1 to 3. We have carefully checked the data again and recalculated as necessary, as detailed below.

**Table 1. Weight Status, Weight Perception, the Number of Confidants, Experience of Being Bullied, and** **the General Health Questionnaire (GHQ)-12 Scores in Boys and Girls.**

|  | **Boys** | **Girls** |
| --- | --- | --- |
| ***N*** | 8,108 | 7,171 |
| **Age (years), mean ± SD** | 15.3 ± 1.7 | 15.3 ± 1.7 |
| **BMI (kg/m^2^), mean ± SD** | 20.2 ± 3.2 | 19.9 ± 2.7 |
| **Weight status, *n* (%)** | | |
| Underweight | 716 (8.8) | 922 (12.9) |
| Low-normal weight | 1,610 (19.9) | 1,765 (24.6) |
| Mid-normal weight | 3,402 (42.0) | 2,882 (40.2) |
| High-normal weight | 1,456 (18.0) | 1,131 (15.8) |
| Overweight | 699 (8.6) | 404 (5.6) |
| Obese | 225 (2.8) | 67 (0.9) |
| **Weight perception, *n* (%)** | | |
| *Too* thin | 589 (7.3) | 87 (1.2) |
| *A bit* thin | 1,610 (19.9) | 328 (4.6) |
| Good | 3,291 (40.6) | 1,732 (24.2) |
| *A bit* fat | 1,910 (23.6) | 3,328 (46.4) |
| *Too* fat | 708 (8.7) | 1,696 (23.7) |
| **Number of confidant(s)^a^, *n* (%)** | | |
| None | 1,844 (22.7) | 777 (10.8) |
| 1 | 744 (9.2) | 860 (12.0) |
| 2 | 1,257 (15.5) | 1,420 (19.8) |
| 3 | 822 (10.1) | 1,136 (15.8) |
| ≥ 4 | 3,441 (42.4) | 2,978 (41.5) |
| **Experience of being bullied (past 1 year), *n* (%)** | | |
| (+) | 621 (7.7) | 505 (7.0) |
| **GHQ-12 score, *n* (%)** | | |
| ≥ 4 | 2,699 (33.3) | 3,936 (54.9) |

*n*, number of subjects; SD, standard deviation.

^a^ How many people are there for you to confide in about your concerns/problems?

Corrections to Table 1:

- The original table made a typo in the number of samples for boys (originally “*8,180*”). We have now corrected the number to be “*8,108*”.
- The original table mistakenly included the numbers of obese subjects in the frequency of overweight adolescents, in addition to stating the obesity group by itself. We have now corrected the numbers and the frequencies for boys (originally “*924 (11.6)*”) and girls (originally “*471 (6.6)*”) to be “*699 (8.6)*” and “*404 (5.6)*”.
- The original table mistakenly excluded 35 boys and 34 girls from the total sample when categorizing them into the five WS categories. We have now corrected the numbers and the frequencies of boys and girls in each WS category to include those who were mistakenly excluded.

According to the corrections in Table 1, the following changes were also made to the text.

Results section, page 10, line 187:

- The original text mistakenly included the numbers of obese subjects in the proportions of overweight boys and girls. We have now corrected the frequencies of overweight samples for boys (originally “*14.2%*”) and girls (originally “*7.5%*”) to be “*11.4%*” and “*6.6%*”.

**Table 2. Weight Perception and the Number of Confidants According to Weight Status, in Boys and Girls, *n* (%).**

|  | **Underweight** | **Low-normal weight** | **Mid-normal weight** | **High-normal weight** | **Overweight** |
| --- | --- | --- | --- | --- | --- |
|  | **Boys (*N* = 8,108)** | | | | |
| **Weight perception** | | | | | |
| *Too* thin | 239 (33.4) | 220 (13.7) | 115 (3.4) | 11 (0.8) | 4 (0.4) |
| *A bit* thin | 262 (36.6) | 586 (36.4) | 673 (19.8) | 78 (5.4) | 11 (1.2) |
| Good | 184 (25.7) | 648 (40.2) | 1,773 (52.1) | 590 (40.5) | 96 (10.4) |
| *A bit* fat | 24 (3.4) | 132 (8.2) | 759 (22.3) | 636 (43.7) | 359 (38.9) |
| *Too* fat | 7 (1.0) | 24 (1.5) | 82 (2.4) | 141 (9.7) | 454 (49.1) |
| **Number of confidants^a^** | | | | | |
| 0–3 | 432 (60.3) | 941 (58.4) | 1,886 (55.4) | 843 (57.9) | 565 (61.1) |
| ≥ 4 | 284 (39.7) | 669 (41.6) | 1,516 (44.6) | 613 (42.1) | 359 (38.9) |
|  | **Girls (*N* = 7,171)** | | | | |
| **Weight perception** | | | | | |
| *Too* thin | 68 (7.4) | 14 (0.8) | 4 (0.1) | 1 (0.1) | 0 (0.0) |
| *A bit* thin | 200 (21.7) | 105 (5.9) | 22 (0.8) | 0 (0.0) | 1 (0.2) |
| Good | 408 (44.3) | 702 (39.8) | 557 (19.3) | 58 (5.1) | 7 (1.5) |
| *A bit* fat | 214 (23.2) | 794 (45.0) | 1,681 (58.3) | 547 (48.4) | 92 (19.5) |
| *Too* fat | 32 (3.5) | 150 (8.5) | 618 (21.4) | 525 (46.4) | 371 (78.8) |
| **Number of confidants^a^** | | | | | |
| 0–3 | 528 (57.3) | 1,008 (57.1) | 1,638 (56.8) | 709 (62.7) | 310 (65.8) |
| ≥ 4 | 394 (42.7) | 757 (42.9) | 1,244 (43.2) | 422 (37.3) | 161 (34.2) |

*n*, number of subjects.

^a^ How many people are there for you to confide in about your concerns/problems?

Corrections to Table 2:

- The original table mistakenly excluded 35 boys and 34 girls from the total sample. We have now corrected the number and the frequencies to include those who were mistakenly excluded, in each cell in the table.
- The original table made typos in the number of samples for boys (originally “*7,671*”) and girls (originally “*6,462*”). We have now corrected these numbers to be “*8,108*” and “*7,171*”. Please note that the values in the original table were calculated among 8,108 - 35 = 8,073 boys and 7,171 - 34 = 7,137 girls.

**Table 3. Odds Ratios and 95% CIs for the Effects of WS and WP on Having Few Confidants When Adjusted for Age, Experience of Being Bullied, and Depression/Anxiety Symptoms.**

|  | **Unadjusted** | **Adjusted** |
| --- | --- | --- |
|  | **Boys** | |
| Underweight | **1.22 (1.04–1.44)** | **1.23 (1.04–1.45)** |
| Low-normal weight | **1.13 (1.00–1.27)** | *1.12 (0.99*–*1.27)* |
| High-normal weight | 1.10 (0.98–1.25) | 1.09 (0.96–1.24) |
| Overweight | **1.26 (1.09–1.47)** | **1.21 (1.04–1.41)** |
| *Too* thin | **1.37 (1.14–1.64)** | **1.21 (1.01–1.45)** |
| *A bit* thin | 1.03 (0.92–1.17) | 1.00 (0.90–1.13) |
| *A bit* fat | **1.33 (1.19–1.49)** | **1.21 (1.07–1.36)** |
| *Too* fat | **1.54 (1.30–1.83)** | **1.31 (1.10–1.56)** |
|  | **Girls** | |
| Underweight | 1.01 (0.87–1.17) | 1.01 (0.86–1.17) |
| Low-normal weight | 1.01 (0.90–1.14) | 1.00 (0.89–1.13) |
| High-normal weight | **1.28 (1.11–1.47)** | **1.26 (1.09–1.46)** |
| Overweight | **1.45 (1.18–1.78)** | **1.41 (1.15–1.74)** |
| *Too* thin | 1.43 (0.92–2.24) | 1.31 (0.83–2.06) |
| *A bit* thin | 1.04 (0.82–1.33) | 0.99 (0.78–1.27) |
| *A bit* fat | **1.15 (1.02–1.29)** | 1.05 (0.93–1.18) |
| *Too* fat | **1.42 (1.24–1.63)** | **1.19 (1.03–1.37)** |

CI, confidence interval; WS, weight status (reference: mid-normal weight); WP, weight perception (reference: perceiving one's weight to be good). Significant associations are indicated in bold (*p* < .05) and italic (*p* < .10) letters. Covariates include age, experience of being bullied, and the GHQ-12 score (0/1).

Corrections to Table 3 (including mistakenly excluded 35 boys and 34 girls):

- We reran the binary logistic regression since the numbers and the frequencies of boys and girls in each WS category were recalculated. Although a few changes in some of the values were observed, adding the adolescents who were mistakenly excluded in the analysis did not significantly affect the results.

According to the corrections in Table 3, the following changes were also made to the text.

Binary logistic regression sub-section, page 14, lines 215–216:

- As a result of rerunning the binary logistic regression, only the *p* value for the association between being overweight and having few confidants was changed, from .01 to .001, as below.

“*In girls, being overweight (p < .001) and high-normal weight (p < .01) and perceiving oneself to be too fat (p < .05) showed significant odds ratios.*”

We have also corrected some details that were missing in the main text as follows.

Populations and procedures sub-section, page 6, lines 100–101:

- The time span of data collection has been corrected as follows: “*A school-based cross-sectional survey targeting junior and senior high school students (Grades 7*–*12) was conducted from 2006 to 2009 in Japan.*”

Binary logistic regression sub-section, page 13, lines 216–218:

- We apologize that we did not mention the missing data when conducting the adjusted binary logistic regression analyses. A few sentences have been added to the Binary Logistic Regression sub-section as below.

“*In the adjusted analyses, 20 boys and 32 girls were excluded due to missing data about the experience of being bullied; a total of 8,088 boys and 7,139 girls were analyzed.*”

Discussion section, page 16, line 252–253:

- We made a minor error for the sentence expression in the original text. We have now corrected the sentence expression as below.

“*Overweight adolescents being more likely to have few confidants is in line with studies in the literature.*”
