# Supplementary material for: The association of weight status and weight perception with number of confidants in adolescents
Source: PLoS One. 2019 Dec 4;14(12):e0225908. doi: 10.1371/journal.pone.0225908 (PMC6892562; doi:10.1371/journal.pone.0225908)
Supplement: Attachment — Submitted filename: Response to Reviewers.docx [file pone.0225908.s006.docx]

Response to Reviewers:

Thank you very much for your constructive comments. We have carefully considered the additional points addressed and have made changes to the manuscript accordingly. In this response letter, original comments by the Reviewers are indicated in **bold**. Our responses are written in regular text. Locations of each part that was revised are indicated in red. Manuscript text is indented and indicated in “*quotations and italics*”, and changes to the text are indicated in *blue*. In the manuscript file, changes to the text are also indicated in blue.

**Response to the comments by the Reviewer 1 (R1)**

**R1 – Comment 1:**

**Authors need to verify the data distribution and must use the appropriate statistical tests. Using other tests can change the Results, and thus the Discussion and Conclusion. How did Authors verify the normal distribution of data? What tests they used? Authors should complete this information.**

**Response:**

Thank you for the feedback. In our analyses, the associations of WS or WP with the number of confidants were tested using binary logistic regression, with WS or WP specified as the independent variable and number of confidants (“Few” vs. “More” confidants) as the dependent variable. The independent variable was entered as a dichotomous variable: being either any of the 4 case groups (“underweight” “low-normal weight” “high-normal weight” or “overweight”) or “normal weight” for WS; having a self-perception of being either any of the 4 case groups (“too thin” “a bit thin” “a bit fat” or “too fat”) or “good” for WP. To our knowledge, logistic regression, unlike linear regression, does not require that the dependent variable or error terms be normally distributed, and verification of the normality may not be required.

If the comment refers to the issue of how well our data fits the logistic regression models which assumes a probability curve of independent variables to be sigmoid shaped, we have now conducted the Hosmer-Lemeshow test, a goodness of fit test for logistic regression. Testing the covariate ‘age’, the only variable that was entered as continuous value, gave *p*-values of 0.43 and 0.75 for boys and girls respectively, indicating no evidence of poor fit. Testing the dichotomous variables gave *p*-value of 1. We have now added these additional analyses in the Materials and methods section as follows.

Statistical analysis sub-section, page 10, lines 185–188:

“*Since age was a continuous variable, the present study conducted the Hosmer-Lemeshow test, a goodness of fit test for logistic regression, to assess how well the data fits the logistic regression models. It gave p-values of 0.43 and 0.75 for boys and girls respectively, indicating no evidence of poor fit. Testing the dichotomous variables gave p-value of 1.*”

To respond to R1 – Comment 1, we have received advice from Dr. Satoshi Usami, an associate professor at the University of Tokyo specializing in statistics, on the normality of the data distribution. He has also given us instruction on the statistical technique for testing the model fit for the logistic regression analyses. We have now listed Dr. Usami in the Acknowledgements as follows.

Acknowledgements section, page 20, lines 336–338:

“*The authors thank Dr. Satoshi Usami at Center for Research and Development on Transition from Secondary to Higher Education, the University of Tokyo, for providing us direct technical help* *and expertise on statistics.*”

**Response to the comments by the Reviewer 2 (R2)**

**R2 – Comment 1:**

**Thank you for responding accordingly to each comment. All of them have been addressed well. Congratulations on your rigorous analysis.**

**Response:**

Thank you for the feedback.

**Response to the comments by the Reviewer 3 (R1)**

**R3 – Comment 1:**

**Introduction: Although the authors agree to remove the sentence “Analyses of the associations are stratified by sex and further adjusted for age, experience of being bulled, and depressive and anxiety symptoms.” Of the Introduction and report deleting it, it still remains in the manuscript in the page 5, lines 95 and 96.**

**Response:**

Thank you for catching out mistake. We apologize for overlooking this in the revised manuscript. We have now removed the sentence from the line 94 in the Introduction section.

**R3 – Comment 2:**

**Methods: 1. I suggest removing the sampling process of the Results Section and including it in the Methodology Section.**

**Response:**

Thank you for the suggestion. We believe that R3 – Comment 2 is based on the sentences, which were originally located in the Results section, as follows: “*In the adjusted analyses, 20 boys and 32 girls were excluded due to missing data about the experience of being bullied; a total of 8,088 boys and 7,139 girls were analyzed.*” We agree that these sentences belong to the Materials and methods section. The sentences have been removed from the line 225 in the Results section and added to the Materials and methods section as follows.

Statistical analysis sub-section, page 10, lines 180–185:

“*Analyses were adjusted for age, experience of being bullied (0 or 1) and GHQ-12 score (0 or 1). Mid-normal weight or perceiving oneself as good was chosen as the reference category in the analyses. All analyses were stratified by sex. In the adjusted analyses, 20 boys and 32 girls were excluded due to missing data about the experience of being bullied; a total of 8,088 boys and 7,139 girls were analyzed.*”

**R3 – Comment 3:**

**Methods: 2. I apologize because I didn’t give the correct information. Was the General Health Questionnaire validated for adolescents? Could you indicate the alpha data? Please complete this.**

**Response:**

Thank you for the comment. The validity of GHQ-12 in adolescents has been confirmed in the review paper (Tait et al. 2002*****). In addition, the validity and reliability of the Japanese version of GHQ-12 have been established in people of all ages (Nakagawa & Daibo 1985*****). The Cronbach’s alpha using the present data was 0.84, applying the bimodal scoring method (0-0-1-1). Regarding the cut-off points for GHQ-12, the score of 4 or higher in the possible score range of 0 to 12 has been reported as indicative of the risk of depression/anxiety in Japanese adults and adolescents (Honda et al. 2001*****). We have now added the above information to the Materials and methods section. Also, to reflect the above details, the 3 studies have been added to the reference list, numbered Reference 34, 35, and 36. One study has been removed (*Doi Y, Minowa M. Factor structure of the 12-item General Health Questionnaire in the Japanese general adult population. Psychiatry Clin Neurosci Blackwell Sci. 2003;57(4):379–83.*) from the reference list, since the study did not examine the use of GHQ-12 in adolescent samples.

*****Additions to the Reference list, pages 25–26, lines 444–450:

*34.	Tait RJ, Hulse GK, Robertson SI. A review of the validity of the General Health Questionnaire in adolescent populations. Aust N Z J Psychiatry. 2002;36(4):550–7. doi: 10.1046/j.1440-1614.2002.01028.x*

*35.	Nakagawa Y, Daibo I. Manual of the Japanese version of the General Health Questionnaire. Tokyo: Nihon Bunka Kagakusha; 1985.*

*36.	Honda S, Shibata Y, Nakane N. Screening of mental disorder with GHQ-12. J Heal Welf Stat. 2001;48(10):5–10.*

Covariates sub-section, pages 9–10, lines 167–176:

“*Depression/anxiety symptoms were measured using the Japanese version of the 12-item General Health Questionnaire (GHQ-12).* *The validity of GHQ-12 in adolescents has been previously confirmed [34].* *In addition, the validity and reliability of the Japanese version of GHQ-12 has been established in people of all ages [35].* *While each item of GHQ-12 was rated on a four-point Likert scale, the present study applied a bimodal scoring method (0-0-1-1); the Cronbach’s alpha using this scoring method was 0.84 in the present sample. A score of 4 or higher in the possible score range of 0 to 12 is indicative of the risk of depression/anxiety in Japanese adults and adolescents [36].* *These scores were entered as a dichotomous variable which were coded to 0 (scores from 0–3) and 1 (4–12) in the analyses.*”
